# Neuroendocrine–Immune Axis in Endometriosis: A Review on How the Nervous System Goes Beyond Pain Perception

**DOI:** 10.3390/biom15111536

**Published:** 2025-10-31

**Authors:** Lingyu Chang, Jing Shan, Dajin Li, Xiaoqiu Wang

**Affiliations:** 1 Shanghai Key Lab of Reproduction and Development, Obstetrics & Gynecology Hospital of Fudan University, Shanghai 200433, China; changlingyu@fudan.edu.cn (L.C.); shanjing0719@163.com (J.S.); 2 Shanghai Key Lab of Female Reproductive Endocrine Related Diseases, Obstetrics & Gynecology Hospital of Fudan University, Shanghai 200433, China

**Keywords:** endometriosis, nerve, endocrine, immunity, hormone

## Abstract

Endometriosis (EMS) is an estrogen-dependent disorder that affects about 10% of reproductive-age women. EMS affects female neuroendocrine and reproductive functions, greatly compromising female reproductive health and quality of life. However, the pathogenesis of EMS has not been fully elucidated, and effective treatment options are still lacking. The neuroendocrine–immune axis can achieve cross-regulation through the interaction of neurotransmitters, hormone-like substances, cytokines, and their receptors to maintain body homeostasis, and this imbalance of regulation is involved in the occurrence and development of EMS. In this narrative review, we summarize recent progress on the role of the neuroendocrine–immune axis in the pathogenesis of endometriosis, as well as the emergence of novel therapies targeting this axis. This review aims to offer a novel perspective for the in-depth exploration of EMS pathogenesis and the identification of potential effective treatments.

## 1. Introduction

Endometriosis (EMS) is a common condition characterized by the presence of endometrial glands and stromal cells outside the uterine cavity, affecting approximately 10% of women in their reproductive age [1]. While the involvement of neuroendocrine and immune factors in endometriosis has been partly clarified, the underlying mechanisms are still unclear. Imbalance of the neuroendocrine–immune axis can abnormally activate hormones and cytokines, disrupt homeostasis, and promote adhesion, implantation, and invasion of ectopic endometrial tissue, leading to ectopic lesion formation. The nervous, endocrine, and immune systems do not exist in isolation; they interact to form a balance or a promoting effect during the pathophysiological process. There is a bidirectional information transmission mechanism between the neuroendocrine system and the immune system. That is, the immune system is regulated by the nervous and endocrine systems and also resizes neurological and endocrine functions. The immune system has abundant neuroendocrine hormones, synthesizing neurotransmitters and endocrine hormones. Cytokines produced by the immune system can affect the central nervous system, and the nervous system can also synthesize cytokines and their specific receptors. Women with endometriosis are often accompanied by great psychological stress, which causes neuroendocrine–immune axis imbalance, expediting disease progression and giving rise to pelvic pain and infertility, resulting in a reduction in their quality of life [2]. Pain in patients with EMS often worsens in a time-dependent manner, indicating the inevitability of neurological abnormalities. There are significant individual differences in pain perception, but they are weakly related to the disease stage [3,4]. Accordingly, minor diseases may be accompanied by severe pain, and vice versa [5]. Elevated stress levels in patients with EMS alter hormone secretion, emotion and behavior, sexual function, and eating habits [6], resulting in an increased incidence of inflammatory complications such as inflammatory bowel disease, fibromyalgia, chronic fatigue, and even autoimmune diseases like thyroid disease, systemic lupus erythematosus, and multiple sclerosis [7,8]. Therefore, an in-depth analysis of the mechanisms of neuroendocrine–immune axis imbalance in EMS is requisite for illustrating the pathogenesis and delivering the theoretical and experimental basis for effective treatment strategies.

However, despite numerous reviews focusing on the pathophysiology of endometriosis from endocrine, immune, or neurological perspectives, most of these studies have addressed each system in isolation. In contrast, our review integrates recent findings on the interactions between the neuroendocrine and immune systems, offering a more holistic understanding of their collective role in the pathogenesis of EMS. This review provides novel insights into how imbalances within this axis affect the clinical manifestations of EMS, offering important implications for advancing therapeutic strategies. By emphasizing the interconnectedness of these systems, our work underscores the significance of considering them together when developing effective treatments and understanding disease mechanisms in endometriosis.

## 2. Bioactive Substances of the Nervous System and Endometriosis

The nerve fibers associated with EMS correlated with the severity of dysmenorrhea. Increased nerve fiber density and nerve hypertrophy in ovarian endometriotic lesions were relevant to pelvic pain [5]. There are multiple small and unmyelinated nerve fibers in the eutopic endometrium of patients with EMS, but not in the endometrium of non-EMS patients [9]. The density of nerve fibers in the endometrium and myometrium of EMS patients is significantly higher than that in women wihout endometriosis, and this increase is closely related to pain [10]. Hormone therapy can significantly reduce the density of nerve fibers in the endometrium and myometrium, thereby alleviating pain [11]. Compared with a healthy peritoneum, the sensory fiber density increases, the sympathetic fiber density decreases in the ectopic lesions in the peritoneal cavity, and the nerve fiber density is higher in the deeply infiltrated endometriosis than in the peritoneal cavity [12]. Gene expression levels associated with nerve fiber growth in the endometrium are upregulated in patients with EMS compared to those without EMS [13]. The eutopic endometrium of EMS patients had higher levels of several neuroactive factors compared with normal endometrium, including neuropeptide Y (NPY), vasoactive intestinal peptide (VIP), and substance P (SP), which may promote nerve fiber growth [9]. Transient receptor potential vanilloid 1 (TRPV1) and transient receptor potential cation channel, subfamily A, member 1 (TRPA1) are highly expressed in patients with EMS, and TNF-α and IL-1β can affect the membrane potential of sensory nerves through a TRPV-1-dependent mechanism, which is related to the occurrence of peripheral nerve pain [14]. TRPV1-positive neurons can induce neurogenic inflammation through the release of substance P (SP) and calcitonin gene-related peptide (CGRP), which participate in the occurrence of EMS-related pain [15].

The expressions of growth hormone-releasing hormone (GHRH) and GHRH-SV1 (splice variant 1 SV1) were markeredly increased in the eutopic endometrium and ectopic lesions compared with normal endometrium, and were higher in advanced (stages III-IV) than in early (stage I-II) endometriosis [16]. It has been clarified that in ectopic lesions, eutopic endometrium, and normal endometrium, SV1 expression is highest in the eutopic endometrium, whereas GHRH expression is highest in ectopic lesions [17]. GHRH and GHRH-SV1 may be involved in the occurrence and development of EMS. GHRH antagonists can inhibit the proliferation and survival of ectopic endometrial cells, reduce the size of ectopic focus in EMS models, and may represent an effective complementary treatment strategy for EMS [17]. The increased levels of growth hormone (GH) in ectopic lesions not only facilitate cell proliferation but also reduce intercellular adhesion and promote cell migration, thus participating in the progression of EMS [18].

The level of somatostatin (SS) is significantly upregulated in the proliferative endometrium of early-stage (I-II) EMS [19]. The expression of SS in ectopic endometrial cells was significantly higher than that of normal controls, and the expression levels of SSTR1-5 (somatostatin receptor 1-5) in eutopic endometrium and ectopic lesions of EMS patients were significantly higher than that of normal endometrium [20]. Studies have found that the SSTR ligand octreotide (somatostatin receptor agonist) and somatostatin analog SOM230 significantly inhibited migration and proliferation in endometrial stromal cells [21,22]. Additionally, the somatostatin analog lanreotide decreased the expression levels of vascular endothelial growth factor (VEGF) and MMP-9 and significantly reduced the volume of ectopic lesions [23].

It has been shown that the amounts of neurons expressing mu-opioid receptors (MORs) decreased significantly in the ventral periaqueductal gray (PAG) in the EMS model, while MOR modulates the activity of PAG and is involved in the occurrence of EMS-related pain [24]. Endomorphin-1 (EM-1) has a high affinity for MOR (mu-opioid receptor). The expression of EM-1 in the hypothalamus, pituitary, and ovary was significantly increased in the rat model of EMS, accompanied by the increased levels of EM-1 and the decreased levels of follicle-stimulating hormone (FSH), luteinizing hormone (LH), estradiol (E2), and progesterone in plasma. EM-1 may be involved in the occurrence of EMS-related infertility by regulating the hypothalamic–pituitary–ovarian axis. Naloxone (MOR antagonist) can significantly reduce the levels of EM-1 in the hypothalamus, pituitary, ovary, and plasma, while increasing FSH and LH levels. These effects suggest that naloxone may be a potential therapeutic agent for EMS-related infertility [25].

**Figure 1:**
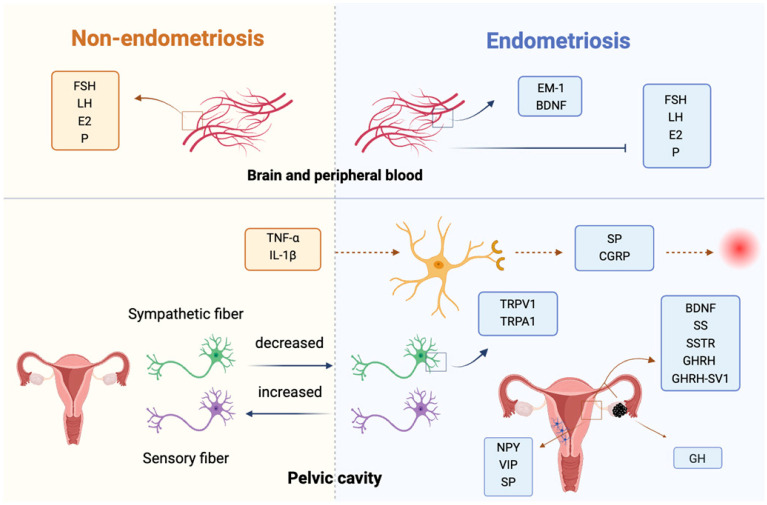
Bioactive substances of the nervous system and endometriosis. In the central nervous system, altered opioid signaling (MOR/EM-1) and neurotrophins (BDNF, NT3) influence pain perception and reproductive hormone regulation. In the peripheral/lesion environment, increased sensory and sympathetic fibers, neuropeptides (NPY, VIP, SP), and ion channels (TRPV1/TRPA1) promote neurogenic inflammation and pain. Local growth factors (GHRH, GH, somatostatin, and VEGF) and cytokines from ectopic lesions further stimulate nerve-immune-endocrine interactions, linking aberrant innervation with pelvic pain and infertility. Abbreviations: EMS, endometriosis; NPY, neuropeptide Y; VIP, vasoactive intestinal peptide; SP, substance P; TRPV1, transient receptor potential vanilloid 1; TRPA1, transient receptor potential cation channel subfamily A member 1; CGRP, calcitonin gene-related peptide; GHRH, growth hormone-releasing hormone; SV1, GHRH splice variant 1; GH, growth hormone; SS, somatostatin; SSTR, somatostatin receptor; MOR, mu opioid receptor; PAG, periaqueductal gray; EM-1, endomorphin-1; FSH, follicle-stimulating hormone; LH, luteinizing hormone; E2, estradiol; P, progesterone; BDNF, brain-derived neurotrophic factor; NT3, neurotrophin 3; GnRH-a, gonadotropin-releasing hormone agonist.

Blood concentrations of brain-derived neurotrophic factor (BDNF) are associated with an increased risk of EMS, and patients with surgically confirmed EMS have higher plasma BDNF concentrations and more pronounced pain symptoms than women without an endometriosis diagnosis (including benign surgical and healthy controls) [26,27]. Compared with endometrium from healthy women, BDNF expression level is higher in the eutopic endometrium of EMS patients [28]. Estrogen can promote the expression of BDNF and neurotrophin 3 (NT3) in ectopic foci [29]. Gonadotropin-releasing hormone agonists (GnRH-a) and progestin can reduce the expression of NTRK2 (BDNF receptor) in deep infiltrating EMS [30], and melatonin could decrease the concentrations of circulating BDNF in EMS patients [31]. Reducing levels of BDNF or its receptors may be beneficial for the treatment of EMS. A schematic representation of this section is shown in Figure 1.

Overall, in EMS, increased nerve fiber density and hypertrophy in ectopic lesions are closely associated with pain severity. Key neuroactive substances, such as NPY, VIP, and SP, promote nerve growth in the eutopic endometrium. Elevated expressions of transient receptor potential channels (TRPV1 and TRPA1) contribute to peripheral nerve pain through neurogenic inflammation. Moreover, the upregulation of GHRH and SS, along with their receptor expressions, facilitates ectopic endometrial cell proliferation and disease progression, highlighting potential targets for therapeutic intervention in pain and inflammation management.

## 3. Neuroendocrine/Neuroimmune System Interactions Are Involved in Endometriosis

It has been validated that there is a correlation between chronic stress and EMS [32]. When the body is stimulated by stressors, the sympathetic nervous system (SNS) and the hypothalamic–pituitary–adrenal (HPA) axis are continuously activated. SNS promotes the secretion and release of peripheral neurotransmitters such as catecholamines. After HPA axis activation, the secretion of corticotropin (CRH) in the paraventricular nucleus of the hypothalamus leads to the release of adrenocorticotropic hormone (ACTH), which acts on the adrenal cortex to regulate the synthesis and secretion of glucocorticoid (GC). Under chronic stress, continuous or repeated activation of the SNS and HPA axis causes neuroendocrine dysfunction. An abnormal increase in GC secretion results in an aberrant immune response and local microenvironment changes, thus promoting the development of EMS. The HPA axis is dysfunctional in patients with EMS. Chronic stress increases the incidence of EMS. Studies revealed that women who have experienced multiple types of abuse over a long period had a 79% increased risk of developing EMS than those who had not experienced any abuse [33]. The typical symptoms of EMS are pain and infertility, followed by sexual discomfort, urination and defecation difficulties. Those critical stressors place EMS patients in a state of chronic stress. When symptoms worsen or the disease is perennial chronic, it will lead to increased levels of self-perceived stress, and patients with EMS exhibit higher levels of anxiety and depression compared with healthy individuals [32]. Chronic stress can also contribute to eating disorders, and the resulting secondary underweight is associated with an elevated risk of EMS [34]. Diet also plays an important role in the establishment and maintenance of EMS, as it may influence inflammation, estrogen activity, the menstrual cycle, and prostaglandin metabolism [35]. Omega-3 fatty acids have an inhibitory effect on the secretion of inflammatory mediators and may play a therapeutic role in the control of EMS- pelvic pain [36].

High levels of CRH induced by chronic stress may contribute to peritoneal inflammation in EMS; CRH blocking agents and interventions that alleviate chronic stress could represrnt therapeutic options for EMS [37]. In the EMS rat model, the number and volume of cystic lesions of rats in the stress group were larger [38]. Chronic psychological stress not only accelerated lesion growth but also enhanced pain sensitivity in EMS-model mice [39]. Under chronic stress, decreased expression of glucocorticoid receptors may reduce glucocorticoid-mediated inhibition of proinflammatory cytokines, resulting in increased levels of these cytokines and the induction of peripheral and central nervous sensitization, which contributes to EMS-related pain [40]. The adrenergic receptor β2 (ADRB2) level in ectopic lesions is higher in mice exposed to chronic stress than in those without stress; meanwhile, the expression of VEGF, proliferating cell nuclear antigen (PCNA), microvessel density, platelet aggregation, and macrophage infiltration are increased in ectopic lesions [41]. The expression level of ADRB2 in ectopic lesions in patients with EMS was higher than that of eutopic and normal endometrium, which was correlated with the rASRM score in EMS [41]. The activation of the ADRB2 signaling pathway participated in neovascularization, increasing cell proliferation, and promoting the growth of ectopic lesions [39].

The hypothalamus–pituitary–gonads (HPG) axis is the main system that regulates reproductive activity. Gonadotropin (GnRH) secreted by the hypothalamus stimulates the pituitary to release FSH and LH, which in turn act on the ovaries to promote follicle development and the synthesis and secretion of sex hormones. Excessive secretion of glucocorticoids (GC) can inhibit GnRH secretion, preventing or delaying follicular development and interfering with ovulation and embryo implantation [42]. The infertility rate of patients with EMS is as high as 30–50%, and chronic stress may be involved in the occurrence of EMS-related infertility. Compared with healthy patients, the serum cortisol levels of women with EMS combined with infertility are significantly higher [43]. Cortisol levels were also significantly higher in the hair of EMS patients [44].

Corticotropin-releasing hormone (CRH) and urocortin (UCN) are neuropeptides that are strongly associated with inflammation and stress, and play a role in the development of EMS. The effects of CRH and UCN are mediated by CRH receptors CRHR1 (CRH-receptors type-1) and CRHR2 (CRH-receptors type-2). The expression of CRHR1 and CRHR2 was highest in ectopic lesions, and the eutopic endometrium has a stronger expression than that of normal endometrium [45]. The levels of CRH, UCN, and CRHR2 in deep infiltrating endometriosis (DIE) lesions were significantly higher than those in ovarian endometrioma (OMA). The expression of COX2 (cyclooxygenase-2) and PLA2G2A (phospholipase-A2 group IIA) in DIE was higher than that in OMA and was positively correlated with CRHR2 expression. UCN can significantly increase COX2 expression in endometrial stromal cells, and this effect can be attenuated by the CRHR2 antagonist astressin-2B, indicating a great activation of inflammatory pathways in DIE [46]. Cyclic adenosine monophosphate (cAMP) and VEGF production are increased due to CRH activating adenylate cyclase, which promotes angiogenesis as well as the implantation and growth of ectopic lesions [38,47].

Thyroid dysfunction with increased thyroid-stimulating hormone (TSH) levels is associated with EMS [48]. TSH acts as a proliferative and pro-oxidative hormone, whereas T3 and T4 could specifically increase the proliferation of ectopic endometrial cells and reactive oxygen species (ROS) production. When thyroid hormone levels increased, the endometriotic implants were larger in a mouse EMS model. In addition, patients with thyroid dysfunction have an increased incidence of chronic pelvic pain and disease scores [49]. The increase in prolactin is involved in the occurrence of EMS. The prolactin levels in the plasma of infertile women with EMS were significantly higher than those without EMS, and the prolactin levels in stage III/IV were higher than in stage I/II EMS patients [50,51]. Women with moderate to severe EMS exhibit dopamine receptor D2 polymorphisms, which may impair post-receptor signal transduction and lead to increased plasma prolactin levels [52]. Prolactin can strongly induce angiogenesis to sustain the growth of ectopic lesions [53]. Patients who received cabergoline (a dopamine agonist) in combination with hormone therapy had significant pain relief [54]. The use of prolactin receptor (PRLR) antibody can completely inhibit the development of EMS in mouse models, withou affecting uterine weight, indicating its potential as an effective therapy for EMS [55]. Studies have shown that the abnormally high expression of the FSH receptor (FSHR) in ectopic focus induces the production of aromatase, which is a key enzyme in E2 production and may be involved in the formation of high levels of estrogen in ectopic lesions [56].

**Figure 2:**
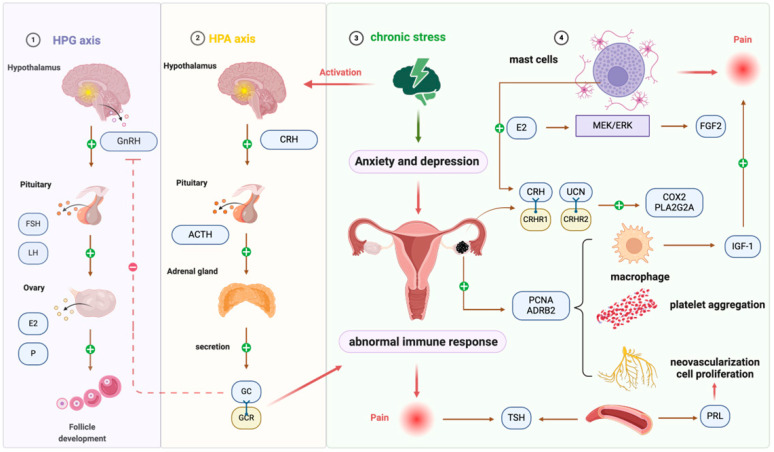
Neuroendocrine/neuroimmune system interactions are involved in endometriosis. Chronic psychological stress persistently activates the HPA axis and SNS, increasing CRH and UCN release as well as ADRB2 signaling. These changes disrupt endocrine homeostasis and immune surveillance, leading to excessive glucocorticoid secretion, abnormal cytokine production, and enhanced angiogenesis. In the peritoneal cavity, mast cells and macrophages are recruited and activated, releasing factors such as IGF-1 and FGF2 that sensitize dorsal root ganglion neurons and aggravate pain perception. This stress-driven crosstalk among neuroendocrine mediators, immune cells, and peripheral nerves contributes to lesion growth, infertility, and chronic pelvic pain in endometriosis. Abbreviations: EMS, endometriosis; SNS, sympathetic nervous system; HPA, hypothalamic–pituitary–adrenal; CRH, corticotropin-releasing hormone; ACTH, adrenocorticotropic hormone; GC, glucocorticoid; HPG, hypothalamus–pituitary–gonads; UCN, urocortin; CRHR2, CRH-receptor type 2; COX2, cyclooxygenase-2; PLA2G2A, phospholipase-A2 group IIA; cAMP, cyclic adenosine monophosphate; VEGF, vascular endothelial growth factor; TSH, thyroid-stimulating hormone; ROS, reactive oxygen species; IGF-1, insulin-like growth factor 1; FGF2, fibroblast growth factor 2; MCs, mast cells; DRG, dorsal root ganglion; ADRB2, β2 adrenergic receptor.

Under chronic stress, the immune system is pivotal in the development of EMS. Animal experiments revealed that chronic stress stimulated the growth of ectopic lesions and increased the recruitment of inflammatory cells, such as mast cells (MCs) and macrophages, in the peritoneal cavity in a mouse model [57]. Chronic stress suppresses the immune system, alters the local immune microenvironment, and changes a variety of immune cells and cytokines in the serum and peritoneal fluid of patients with EMS. These changes reduce immune surveillance, recognition, and destruction of ectopic endometrial cells, thereby promoting the implantation and growth of ectopic endometrial tissue [32,38,57]. Compared with healthy women, the number and activity of macrophages in the peritoneal fluid and eutopic endometrium of patients with EMS increased, and the expression of proinflammatory factors increased significantly. Macrophage-derived insulin-like growth factor 1 (IGF-1) is a nerve-sensitizing factor that can directly innervate diseased nerves related to EMS-associated pain [58]. MCs originate from pluripotent stem cells in the bone marrow and are widely distributed in various tissues throughout the body, especially around blood vessels and nerve endings. Most of the MCs are distributed around the nerve fibers in the ectopic lesions and are mainly involved in the pathogenesis of EMS-related pain. Estrogen promotes the secretion of fibroblast growth factor 2 (FGF2) in MCs through the MEK/ERK pathway, which aggravates the pain symptoms of EMS. Targeted inhibition of the FGF2 receptor can significantly inhibit neurite growth and calcium inflow of dorsal root ganglion (DRG) cells and reduce pain [59]. Mice undergoing chronic stress have increased mast cell infiltration in ectopic lesions [38], and MCs may serve as a potential source of CRH and UCN in peripheral tissues [60]. It should be noted that subtle or deeply infiltrating endometriotic lesions may be under-detected using conventional 2D laparoscopic visualization, contributing to continuous neuroimmune activation and pain. Advanced intraoperative imaging techniques, such as real-time 3D endoscopy combined with near-infrared indocyanine green (ICG) fluorescence, have been shown to enhance lesion detection and may help reduce residual disease and associated pain [61]. This schematic illustrates the structure of this component, as detailed in Figure 2.

Chronic stress disrupts the neuroendocrine and immune balance in EMS, activating the sympathetic nervous system and HPA axis, leading to abnormal glucocorticoid secretion and immune dysfunction. Elevated CRH, UCN, and ADRB2 signaling promote inflammation, angiogenesis, and ectopic lesion growth, while thyroid and prolactin dysregulation further exacerbate disease progression. Immune alterations, including increased macrophage and mast cell activity, contribute to reduced immune surveillance, enhanced ectopic implantation, and nerve sensitization, linking stress, hormonal, and immune pathways to pain and infertility. These interactions underscore the central role of neuroendocrine–immune crosstalk in EMS pathophysiology.

## 4. The Neuroendocrine–Immune Axis Is Involved in the Pathogenesis of Endometriosis

The neuroendocrine–immune axis adjusts the activity of immune cells through releasing neurotransmitters in the nervous system or alteration of endocrine activity, and the cytokines and hormone-like substances secreted by immune cells feedback on the neuroendocrine system. The bidirectional regulation between the neuroendocrine and immune system interact with each other to maintain organismal homeostasis. As an estrogen-dependent disease, EMS is mainly regulated by female reproductive hormones with disturbances in the self-stability regulation of the neuroendocrine–immune axis, resulting in elevated estrogen levels, progesterone resistance, and the concomitant abnormal expression of CRH, ACTH, and UCN [62]. Estrogen can promote proliferation, adhesion, angiogenesis, inflammation, and anti-apoptosis in endometrial epithelial cells (EECs) and ESCs [63]. Progesterone resistance produces excess E2 and overexpression of estrogen receptor β (ER-β) and promotes endometriotic invasion [64]. In addition, dysregulated progesterone receptor A (PR-A) and B (PR-B) ratios and impaired expression of downstream targets disrupt progesterone signaling [65]. Such molecular defects provide a plausible explanation for the reduced responsiveness to progestin therapy observed in patients. Clinically, randomized trials and meta-analyses have demonstrated only limited efficacy of progesterone analogs in alleviating endometriosis-associated pain, underscoring that progesterone resistance represents not only a mechanistic hallmark but also a clinically relevant therapeutic challenge [66]. Patients with endometriosis have higher estrogen levels in menstrual blood and peritoneal fluid than healthy women [67]. Estrogen plays an essential role in EMS-related pain. Estrogen-induced inflammation can stimulate and sensitize peripheral nerves, inducing pain. MCs can release nerve growth factor (NGF) under estrogen stimulation to sensitize dorsal root ganglion cells [68]. Estrogen mediates the interaction between macrophages and nerve fibers in EMS [69]. Estrogen receptors ER-a and ER-β are significantly overexpressed in macrophages of patients with EMS, and 17β-estradiol can increase the expression of ER-α on the macrophage surface and upregulate the production of IL-6 and TNF-α in LPS-activated macrophages [70]. Macrophages are an important source of high levels of IL-6 in the peritoneal fluid of EMS patients, and estrogen/ERα/IL-6-mediated interactive dialog participates in the initiation of EMS [71]. Both ER-α and ER-β were expressed on uterine-related DRG neurons [72]; estrogen activation of peripheral sensory neurons can enhance pain caused by inflammatory mediators [73]. Plasma levels of progesterone in patients with EMS were decreased and associated with disease progression and pain, accompanied by an increased rate of CD56^+^ PR^+^ and CD8^+^PR^+^ abdominal lymphocytes during advanced stages of EMS. High psychological stress leads to an increase in peripheral CRH production and promotes TNF/IL-10 expression. Therefore, progesterone derivatives, CRH blockers, and improved stress response may contribute to the treatment of EMS [37].

**Figure 3:**
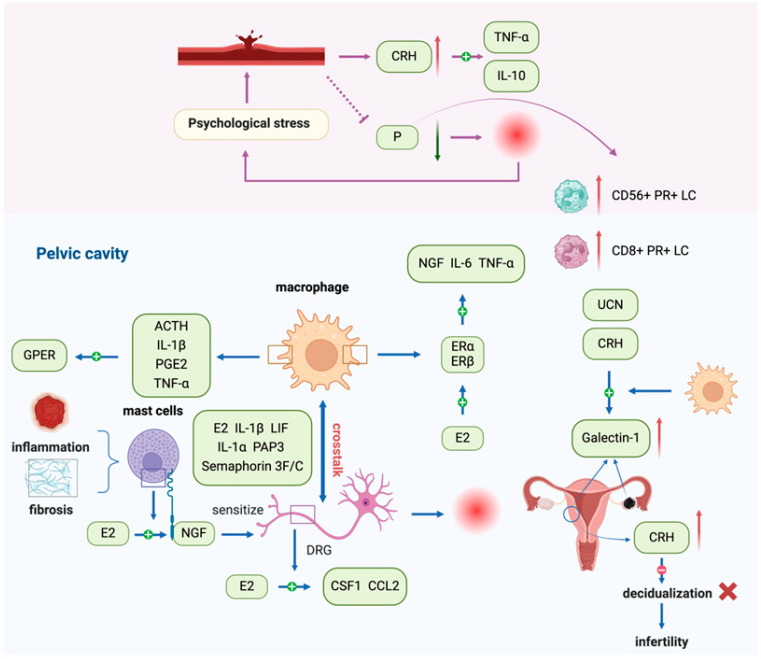
The neuroendocrine–immune axis is involved in the pathogenesis of endometriosis. The neuroendocrine–immune axis regulates immune cell activity via neurotransmitters and endocrine mediators, while immune-derived cytokines and hormone-like factors feed back to the neuroendocrine system. Dysregulation of this axis in endometriosis (EMS) leads to elevated estrogen, progesterone resistance, and abnormal CRH/ACTH/UCN expression, which together promote proliferation, adhesion, angiogenesis, inflammation, and pain. Estrogen-induced NGF release from mast cells sensitizes DRG neurons, while macrophage–nerve interactions mediated by cytokines (e.g., IL-6, IL-1β, LIF, PAP3) and exosomal miR-21-5 drive neurogenesis and immune infiltration. Stress-related CRH and ACTH further amplify macrophage activity through GPER, and galectin-1 upregulation links stress and infertility. Abbreviations: EMS, endometriosis; CRH, corticotropin-releasing hormone; ACTH, adrenocorticotropic hormone; UCN, urocortin; EECs, endometrial epithelial cells; ESCs, endometrial stromal cells; ER-α, estrogen receptorα; ER-β, estrogen receptor β; NGF, nerve growth factor; DRG, dorsal root ganglion; LIF, leukemia inhibitory factor; PAP3, pancreatitis-associated protein 3; M-CSF, macrophage colony-stimulating factor; CCL2, chemokine ligand 2; PGE2, prostaglandin E2; GPER, G protein-coupled estrogen receptor.

Clinical symptoms such as pain and infertility due to EMS can produce stress, and continued exposure to this stress response may seriously affect the endocrine and immune systems. The high expression of CRH in the endometrium of patients with EMS may disrupt the decidualization process, contributing to infertility, while sustained activation of mast cells promotes inflammation and fibrosis [74,75]. Estrogen can induce macrophages to secrete diversified NGFs. A recent study demonstrated that targeting NGF therapy significantly reduced pain sensitivity in a mouse model of endometriosis, whereas inhibition of VEGFR1 and BDNF did not have analogous effects [76]. The coexistence and proximity of macrophages and nerve fibers in ectopic lesions suggest that macrophages are involved in neurogenesis [77]. Nerve fibers can recruit macrophages to migrate toward the ectopic lesions. Estrogen and many cytokines, including LIF, IL-1α, IL-1β, and pancreatitis-associated protein 3 (PAP3),mediating the migration of macrophages to nerve fibers [29]. After phagocytosis by macrophages, the neuron-derived exosome miR-21-5 can mediate its infiltration into peripheral nerves, and estrogen can also stimulate peripheral nerves to secrete CSF1 (macrophage colony-stimulating factor, M-CSF) and CCL2, promoting the migration of macrophages to ectopic lesions [29]. Macrophages express signal proteins Semaphorin 3F and 3C, which are involved in sympathetic denervation in EMS [78]. The expression of G protein-coupled estrogen receptor (GPER) was upregulated in EMS-associated macrophages, and the stress-related factor ACTH and inflammatory factors IL-1, TNF, and PGE2 secreted by macrophages could regulate the expression of GPER, suggesting that estrogen enhanced the functional regulation of macrophages in EMS [79]. Galectin-1 is highly expressed in ectopic foci compared with eutopic endometrium, and its expression is higher in eutopic endometrium than in normal endometrium. CRH and UCN can upregulate the expression of galectin-1 in macrophages, which is involved in the pathology of EMS and infertility [80]. Figure 3 depicts the schematic of this part.

The neuroendocrine–immune axis critically regulates EMS by mediating interactions between hormones, cytokines, and immune cells. Estrogen promotes proliferation, angiogenesis, inflammation, and nerve sensitization, while progesterone resistance and elevated CRH/ACTH/UCN levels exacerbate disease progression. Macrophages interact closely with nerve fibers under estrogen and cytokine signaling, contributing to neurogenesis, inflammation, and fibrosis. Dysregulation of these pathways affects pain, infertility, and local immune responses. Overall, the bidirectional communication between neuroendocrine signals and immune cells underpins EMS pathophysiology, highlighting potential targets for interventions aimed at restoring hormonal balance, modulating immune activity, and alleviating disease-related symptoms.

## 5. New Treatment Strategies for Endometriosis Through Modulation of the Neuroendocrine–Immune Axis

Chronic stress is involved in the occurrence and development of EMS, and patients are more likely to be inclined toward a chronic stress state. Therefore, in addition to traditional drug and surgical treatments, the treatment of EMS should focus on the patient’s chronic stress state. It is imperative to take reasonable stress management measures promptly, including physiological and psychological intervention measures, to reduce the incidence of EMS, alleviate symptoms, and improve the quality of life. Ameliorating the perceived stress can normalize the cortisol levels to repair their physical functioning [81]. A rich environment is safe and effective in reducing the stress of EMS patients. Studies have indicated that compared with the non-environment-enriched group, the area of ectopic lesions in the EMS model mice of the environmental enrichment group was significantly reduced, accompanied by a significant decrease in CRH and glucocorticoid receptor (GR) expression [82]. Reducing the severity of EMS through chronic stress management has been supported in animal studies. Physical activity can alleviate the pain sensitivity in the EMS mouse model by enhancing the expression of μ-opioid receptors [83]. β-blockers can attenuate the accelerated lesion proliferation and increased pain sensitivity in EMS model mice caused by chronic stress [39].

GnRH-a is a commonly used clinical agent for the treatment of EMS. It effectively relieves painful symptoms but may generate menopausal side effects, sometimes necessitating add-back therapy to prevent bone loss or other symptoms of estrogen deficiency [84]. Surgery combined with leuprorelin or Mirena IUD combined with GnRH-a treatment could reduce LH, FSH and E2 levels, downregulate plasma inflammatory factors, improve ovarian function, lower recurrence rates, and increase pregnancy rates in patients with EMS [85,86,87]. Leuporeline injection can also reduce PRL levels [88].

Oral GnRH antagonists are modern treatments that have emerged in recent years. They can rapidly reduce gonadal steroid hormone levels, avoiding the initial stimulation phase of GnRH agonists. The benefits of GnRH antagonists over GnRH agonists include rapid initiation and reversible action. Elagolix, Relugolix, Linzagolix (Yselty), SKI2670, and SHR7280 are all novel oral non-peptide GnRH antagonists [89,90,91,92,93], Elagolix, Relugolix, and Linzagolix (Yselty) can be used to treat EMS-related pain and are well tolerated [89,90,91,94]. SKI2670 attenuates the growth of ectopic foci in rat models without bone loss [92]. Patients taking Relugolix have an earlier menstrual recovery, which may be more favorable for those who need to prepare for pregnancy after treatment [95]. Relugolix in combination with add-back therapy significantly alleviated dysmenorrhea and non-menstrual pelvic pain while reducing the risk of low estrogen-related side effects [96].

Elinzanetant, a non-hormonal, oral dual neurokinin-1,3 receptor (NK1R, NK3R) antagonist, has been proven to reduce plasma levels of LH, estradiol, and luteal progesterone in a dose-dependent manner, which can be used for the treatment of EMS [97]. Treatment with antalarmin (corticotrophin-releasing hormone receptor type 1 antagonist, CRHR1 antagonist) immensely reduces the volume and number of ectopic lesions in the EMS rat model. CRHR1 antagonists inhibited the increase in proinflammatory cytokines, myeloperoxidase, and inflammatory transcription factors, as well as preventing an increase in CRH and CRHR1 in the tissues of the ectopic foci, which is expected to be a promising therapeutic method for EMS [47].

The schematic of this part is shown in Table 1.

## 6. Conclusions and Perspectives

The neuroendocrine–immune axis is involved in the occurrence and development of endometriosis. Since imbalance among the neuro, endocrine, and immune systems drives the progression of EMS, restoring their fine regulation is an effective treatment strategy. New oral formulations have emerged that demonstrate promising efficacy while simultaneously mitigating adverse effects. However, the high recurrence rate and the improvement of pain and infertility symptoms are still important issues that need continued attention. Future studies should test whether selective modulation of adrenergic signaling, targeting CRH/UCN pathways in immune cells, or combined intervention of neuroendocrine–immune nodes can reduce lesion growth, pain, and infertility.

Exploring the molecular mechanism of neuroendocrine–immune axis dysfunction in EMS is helpful to deepen our understanding of the pathogenesis of EMS and to seek accurate and effective treatment from a new perspective. Targeting the neuroendocrine or neuroimmune dual or triple systems can control the disease while improving EMS-related symptoms such as pain and infertility, improving the quality of life of patients with EMS, and effectively preventing disease recurrence.

## Figures and Tables

**Table 1:** New treatment strategies for endometriosis that modulate the neuroendocrine–immune axis.

Strategy	Mechanism	Efficacy	Safety (Side Effects/ Tolerability)	Level of Evidence	Reference
β-blockers	Antagonistic the activation of β receptors	Inhibit lesion proliferation and reduce pain sensitivity	Not available	Animal study	[39]
Antalarmin (CRHR1 antagonist)	Inhibit the increase of proinflammatory cytokines, myeloperoxidase, inflammatory transcription factors and CRH, CRHR1	Reduce the volume and number of ectopic lesions	Not available	Animal study	[47]
Alleviating the perceived stress	Normalize the cortisol levels	Repair physical functioning	Safe, well-tolerated by participants	Clinical	[81]
Enriched environment	Significant decrease CRH and GR expression	Significantly diminished the area of ectopic lesions	Not available	Animal study	[82]
Physical activity	Enhancing the expression of μ opioid receptors	Release pain	Not available	Animal study	[83]
Surgery combined with leuprorelin/Mirena IUD combined with GnRH-a	Reduce LH, FSH and E2 levels, downregulates the levels of plasma inflammatory factors, reduce PRL levels	Improves ovarian function, reduces the recurrence rates, and increases the pregnancy rate	No significant adverse effects reported	Clinical	[85,86,87,88]
GnRH agonist	Reduce LH, FSH and E2 levels	Relieving painful symptoms	Bone loss, Long-term risk	Clinical (review)	[93]
GnRH antagonists (Elagolix, Relugolix, Linzagolix (Yselty), SKI2670 and SHR7280)	Rapidly reduce FSH, LH and E2 levels	Attenute the growth of ectopic foci, relieve dysmenorrhea and pelvic pain	Elagolix: Hot flush, increased lipids and decreased bone mineral density Relugolix: psychiatric symptoms Linzagolix: headaches and hot flushes SKI2670: Well tolerated SHR7280: oligomenorrhea and increased alanine aminotransferase levels	Clinical; SKI2670 (preclinical)	[89,90,91,92,93,94,96]
Elinzanetant (dual (NK1R, NK3R) antagonist	Reduce levels of LH, E2 and luteal progesterone	No obvious clinical changes	No significant adverse effects reported	Clinical	[97]

## Data Availability

Not applicable.

## Abbreviations

The following abbreviations are used in this manuscript:

EMS, endometriosis; NPY, neuropeptide Y; VIP, vasoactive intestinal peptide; SP, substance P; TRPV1, transient receptor potential vanilloid 1; TRPA1, transient receptor potential cation channel, subfamily A, member 1; CGRP, calcitonin gene-related peptide; GHRH, growth hormone-releasing hormone; GHRH-SV1, growth hormone-releasing hormone splice variant 1 SV1; GH, growth hormone; SS, somatostatin; SSTR, somatostatin receptor; MORs, mu opioid receptors; PAG, periaqueductal gray; EM-1, endomorphin-1; FSH, follicle-stimulating hormone; LH, luteinizing hormone; E2, estradiol; P, progesterone; BDNF, brain-derived neurotrophic factor; NT3, neurotrophin 3; GnRH-a, gonadotropin-releasing hormone agonists; SNS, sympathetic nervous system; HPA, hypothalamic–pituitary–adrenal; CRH, corticotropin; ACTH, adrenocorticotropic hormone; GC, glucocorticoid; ADRB2, β2 adrenergic receptor β2; PCNA, proliferating cell nuclear antigen; HPG, hypothalamus–pituitary–gonads; GnRH, gonadotropin; GC, glucocorticoids; CRH, corticotropin releasing hormone; UCN, urocortin; CRHR2, CRH-receptors type-2; DIE, deep infiltrating endometriosis; OMA, ovarian endometrioma; COX2, cyclooxygenase-2; PLA2G2A, phospholipase-A2 group IIA; cAMP, cyclic adenosine monophosphate; VEGF, vascular endothelial growth factor; TSH, thyroid-stimulating hormone; ROS, reactive oxygen species; PRLR, prolactin receptor antibody; FSHR, FSH receptor; IGF-1, insulin-like growth factor 1; MCs, mast cells; FGF2, fibroblast growth factor 2; EECs, endometrial epithelial cells; ESCs, endometrial stromal cells; ER-β, estrogen receptor β; NGF, nerve growth factor; DRG, dorsal root ganglion; PR, progesterone receptor; PAP3, pancreatitis associated protein 3; M-CSF, macrophage colony stimulating factor; GPER, G protein coupled estrogen receptor; ACTH, adrenocorticotropin; UCN, urocorticotropin; GR, glucocorticoid receptor; NK1,3R, neurokinin-1,3 receptor.
